# A study of antioxidant activity, enzymatic inhibition and in vitro toxicity of selected traditional sudanese plants with anti-diabetic potential

**DOI:** 10.1186/1472-6882-14-149

**Published:** 2014-05-07

**Authors:** Yasmin Hilmi, Muna F Abushama, Haidar Abdalgadir, Asaad Khalid, Hassan Khalid

**Affiliations:** 1 Khartoum College of Medical Sciences, P.O Box 10995, Khartoum, Sudan; 2 Medicinal and Aromatic Plants Research Institute, National Centre for Research, P.O.Box 2420 Khartoum, Sudan

**Keywords:** Diabetes mellitus, Medicinal plants, Antioxidant activity, Glycogen phosphorylase, Brine shrimp

## Abstract

**Background:**

Diabetes mellitus is a chronic metabolic disease with life-threatening complications. Despite the enormous progress in conventional medicine and pharmaceutical industry, herbal-based medicines are still a common practice for the treatment of diabetes. This study evaluated ethanolic and aqueous extracts of selected Sudanese plants that are traditionally used to treat diabetes.

**Methods:**

Extraction was carried out according to method described by Sukhdev *et. al*. and the extracts were tested for their glycogen phosphorylase inhibition, Brine shrimp lethality and antioxidant activity using (DPPH) radical scavenging activity and iron chelating activity. Extracts prepared from the leaves of *Ambrosia maritima,* fruits of *Foeniculum vulgare* and *Ammi visnaga,* exudates *of Acacia Senegal,* and seeds of *Sesamum indicum* and *Nigella sativa*.

**Results:**

*Nigella sativa* ethanolic extract showed no toxicity on Brine shrimp Lethality Test, while its aqueous extract was toxic. All other extracts were highly toxic and ethanolic extracts of *Foeniculum vulgare* exhibited the highest toxicity. All plant extracts with exception of *Acacia senegal* revealed significant antioxidant activity in DPPH free radical scavenging assay*.*

**Conclusions:**

These results highly agree with the ethnobotanical uses of these plants as antidiabetic. This study endorses further studies on plants investigated, to determine their potential for type 2 diabetes management. Moreover isolation and identification of active compounds are highly recommended.

## Background

Diabetes mellitus (DM) is a disease with severe complications and major health/economic impacts. It is a leading cause of morbidity and mortality worldwide, with an estimated 346 million adults being affected in year 2011. WHO projects that diabetes death will increase by two- thirds between 2008 and 2030 [1]. The prevalence of diabetes for all age-groups worldwide was estimated to be 2.8% in 2000 and 4.4% in 2030 [2].

In Sudan diabetes is an increasingly important problem, being responsible for 10% of hospital admissions and mortality [3]. Recently, an increase in incidence of DM has been observed especially among urbanized population indicating that diabetes mellitus is emerging as an important health problem [4]. The results of a small-scale study carried out in 1996 indicated that diabetes population in Sudan is at around one million, 90% of them have type 2 diabetes. It also showed a prevalence of 3.4% of type 2 DM [5].

Nature is an extraordinary source of medicines. The use of traditional medicines and medicinal plants in most developing countries as therapeutic agents for the maintenance of good health has been widely observed. The World Health Organization estimated that 80% of the populations of developing countries rely on traditional medicines, mostly plant drugs, for their primary health care needs. Diabetes is an example of a disease that has been treated with plant medicines. Research conducted in the last few decades on plants used traditionally for treatment of diabetes has shown antidiabetic properties [6].

To date, more than 1200 flowering plants have been claimed to have antidiabetic properties. Among them, only one-third have been scientifically studied and documented in around 460 publications [7].

The Sudanese flora has a vast variety of medicinal plants, which are traditionally used for their antidiabetic property. However, careful assessment including sustainability of such herbs, seasonal variation in activity of phyto-constituents, metal contents of crude herbal anti-diabetic drugs, thorough toxicity study and cost effectiveness is required for their popularity. These efforts may justify the role of novel traditional medicinal plants having anti-diabetic potentials.

Herbal drugs are considered free from side effects than synthetic one. They are less toxic, relatively cheap and popular [8]. However cytotoxicity of these plants needs to be monitored.

Brine shrimp bioassays (BSLT) offer a quick, simple and cost-efficient way of testing the toxicity of plant extracts. It has been developed for screening, fractionation and monitoring of biologically active natural products [9,10].

Oxidative stress has been implicated in the development of many pathophysiological conditions including diabetes. Oxidative stress takes place due to the disturbance of the balance between the formation of reactive oxygen species (ROS) and the defense provided by cellular antioxidants. Medicinal plants provide a natural source of antioxidants that have been used worldwide for treatment of many diseases [6].

The inhibition of glycogen phosphorylase has been used as one method for treating type 2 diabetes. Since glucose production in the liver has been shown to increase in type 2 diabetes patients, inhibiting the release of glucose from the liver’s glycogen’s supplies appears to be a valid approach [11,12].

In the present study we assessed the antioxidant and hypoglycemic activities of ethanolic and aqueous extracts of *Ambrosia maritima, Ammi visnaga, Acacia senegal, Sesamum indicum, Nigella sativa, Foeniculum vulgare* of Sudanese origin. Screening of toxicity of these plants using brine shrimp lethality test, is also investigated.

## Methods

### Plant Material

Plants collected from Khartoum local market, identified and authenticated by Dr. Haider Abdelgadir, Herbarium Curator. Herbarium material was deposited at The Medicinal & Aromatic Plants Research Institute (MAPRI), Khartoum, Sudan. Table 1 shows the tested plants, their parts used and medicinal uses.

**Table 1:** Tested plants

**Plant name**	**Family**	**Parts used**	**Reported medicinal uses**[17-20]
*Acacia senegal*	Fabaceae–Mimosoidae	Fruits	Treatment of diabetes and chronic renal failure. Stem exudates (gums) are used as demulcents and against diarrhoea and ulcers
*Ambrosia maritima*	Asteraceae	Leaves	Hepatoprotective and Molluscicidal, The herbs are used in treatment of urinary tract infections and elimination of kidneystones, whereas the leaves are used as anti-diabetic and anti-hypertensive
*Ammi visnaga*	Apiaceae	Fruits	Used for renal urethra stones, smooth muscle relaxant
*Foeniculum vulgare*	Apiaceae	Fruits	Carminative, flatulence and digestive. It is also used in veterinary medicine
*Nigella sativa*	Ranunculaceae	Seeds	Treatment of diabetes, hypertension, abdominal ulcers, prostate gland and lung injury(Cuneyt Tayman)
*Sesamum indicum*	Pedaliaceae	Seeds	Treatment, for cough and cold. also used as nutritive, laxative, demulcent and emollient propertie

### Preparation of plant extracts

Extraction was carried out according to method described by Sukhdev *et. al.*[13].

### Preparation of ethanolic extract

Specific weight of each plant sample was extracted by soaking in 96% ethanol for about seventy two hours with daily filtration and evaporation. Solvent was evaporated under reduced pressure to dryness using rotary evaporator apparatus and the extract combined together. The yield percentage was calculated as followed:

Weightofextract/weightofsample×100

### Preparation of the aqueous extract

About 50–100 g of each plant sample was soaked in 500 ml hot distilled water, and left till cooled down with continuous stirring at room temperature. Extract was then filtered and freeze dried. Yield percentage was calculated.

Weightofextractobtained/weightofplantsample×100

### Brine Shrimp Lethality Test

*Artemia salina* (shrimp eggs) was placed in natural sea water, and eggs hatched within 48 hrs, providing a large number of larvae (nauplii). The tested sample (20 mg) was dissolved in 2 ml of ethanol. From this solution 5, 50 and 500 μl were transferred to vials (triplicate for each concentration), forming concentrations of 10, 100 and 1000 μg/ml respectively. The solvent was allowed to evaporate overnight. Volume was made to 5 ml with seawater. 10 larvae were placed in each vial using a Pasteur pipette. Vials were incubated at 25–27°C for 24 hrs under illumination. Etoposide (7.4625 μg/ml) was used as positive control, and number of survived larvae were counted. Data was analyzed by Finney Probit Analysis computer program to determine LC_50_ values with 95% confidence intervals [9].

### Antioxidant activity assays

1. DPPH radical scavenging assay

  The DPPH radical scavenging was determined according to the modified method of Shimada *et al.*[14] in 96-wells plate, the test samples were allowed to react with 1,1-Diphenyl-2-picryl-hydrazyl stable free radical (DPPH) for half an hour at 37˚C. The concentration of DPPH was kept as 300 μM. The test samples were dissolved in DMSO while DPPH was prepared in ethanol. After incubation, decrease in absorbance was measured at 517 nm using multiple reader spectrophotometer. Percentage radical scavenging activity by samples was determined in comparison with a DMSO treated control group. All tests and analysis were run in triplicate. Vitamin C was used as positive control

2. Iron chelating activity assay

  The iron chelating ability was determined according to the modified method of Dinis *et al.*[15]. The Fe^+2^ were monitored by measuring the formation of ferrous ion-ferrozine complex. The experiment was carried out in 96 microtiter plate. The plant extracts were mixed with FeSO_4_. The reaction was initiated by adding 5 mM ferrozine. The mixture was shaken and left at room temperature for 10 min. The absorbance was measured at 562 nm. EDTA was used as standard, and DMSO as control. All tests and analysis were run in triplicate.

### Glycogen phosphorylase enzyme assays

Glycogen phosphorylase a (from rabbit muscle), glycogen, glucose-1-phosphate, malachite green, and ammonium molybdate were purchased from the Sigma–Aldrich Corporation. Reagents and solvents were obtained from commercial suppliers and used without further purification. Solvents used were AR grade. The enzymatic inhibition of phosphorylase activity was monitored using multiskan spectrum (Thermo-Scientific) based on the published methods. In brief, GPa activity was measured in the direction of glycogen synthesis by the release of phosphate from glucose-1-phosphate. Each compound was dissolved in DMSO and diluted at different concentrations for IC50 determination. The enzyme was added into the 100 μL buffer with compounds dissolved in containing 50 mM Hepes (pH 7.2), 100 mM KCl, 2.5 mM MgCl2, 0.5 mM glucose-1-phosphate, and 1 mg/mL glycogen in 96-well microplates (costar). After the addition of 150 μL of 1 M HCl containing 10 mg/mL ammonium molybdate and 0.38 mg/mL malachite green, reactions were run at 25°C for 20 min. And then the phosphate absorbance was measured at 620 nm [16].

## Results and discussion

Different medicinal systems that have been discovered as natural hypoglycemic medicine came from the virtue of traditional knowledge and have been used in many countries [7,21,22].

Many herbal extracts are currently traditionally used in Sudan for the treatment of diabetes. However, such medicinal plants have not gained much importance as medicines due to the lack of sustained scientific evidence. In the present study, aqueous and ethanolic extracts of six indigenous antidiabetic medicinal plants from Sudan were studied. The rationale for performing extractions from polar to non-polar solvents is to confirm and validate the inhibitory activity in the aqueous extractions performed in the traditional manner as well as to search for newer, more potent inhibitory compounds in the organic solvents. Since the Sudanese population has long been using all these plants for food and medicinal purposes, they form a part of the local pharmacopoeia.

Increasing evidence in both experimental and clinical studies suggests that oxidative stress plays a major role in the pathogenesis of both types of DM. Free radicals are formed disproportionately in diabetes by glucose oxidation, nonenzymatic glycation of proteins, and the subsequent oxidative degradation of glycated proteins. Abnormally high levels of free radicals and the simultaneous decline of antioxidant defense mechanisms can lead to damage of cellular organelles and enzymes, increased lipid peroxidation, and development of insulin resistance. These consequences of oxidative stress can promote the development of complications of DM. Changes in oxidative stress biomarkers, and use of antioxidants is an important trend in the treatment of DM [23].

In the present work we studied the anti-oxidant activity of ethanolic and aqueous extracts of investigated plants. Samples will be considered to have high or significant antioxidant capacity with IC50 < 50 μg/ml (extract) or IC50 < 10 μg/ml (compounds), moderate antioxidant capacity with 50 < IC50 < 100 μg/ml (extract) or 10 < IC50 < 20 μg/ml (compounds) and low antioxidant capacity with IC50 > 100 μg/ml (extract) or IC50 > 20 μg/ml (compounds) [24]. *Ambrosia maritima, Ammi visnaga* and *Foeniculum vulgare* exhibited high antioxidant activity in DPPH free radical scavenging assay with IC50 of 36 μg/ ml of ethanol extract of *Ambrosia maritima*, 41 μg/ ml of ethanol extract of *Ammi visnaga,* 47 μg/ml of aqueous extract of *Ammi visnaga,* 49 μg/ml of ethanol extract of *Foeniculum vulgare* and 31 μg/ml of aqueous extract of *Foeniculum vulgare.* This may support the traditional usage of these plants to improve complications such oxidative stress that caused by DM as well as many other diseases. A study in Egypt by Abu Zid and coworkers showed a moderate antioxidant activity of aqueous extract of *M. ambrosia*[25]. Results of *Achyranthes aspera* extracts in alloxan-treated mice revealed significant anti-hyperglycemic activity that may be mediated by diminished oxidative stress [26]. A study of *Eucalyptus globulus*, *Salvia officinalis* growing in Algeria and *Guiera senegalensis* growing in Sudan demonstrated that the 96% alcoholic leaf extracts had a significant blood-glucose lowering potential in glucose loaded rats with minimum toxicity [27]. Swanston and coworkers reported that agrimony, alfalfa, coriander, eucalyptus and juniper, can retard the development of streptozotocin diabetes in mice [28]. In another study, ethanolic crude extract of *Sorbus decora* demonstrates both anti-hyperglycemic and insulin-sensitizing activity *in vivo*, thereby confirming anti-diabetic potential and validating traditional medicine [29]. *Trigonella foenum-graecum*, *Atriplex halimus*, *Olea europaea*, *Urtica dioica*, *Allium sativum*, *Allium cepa*, *Nigella sativa*,and *Cinnamomum cassia* were tested for their antidiabetic properties. Results indicated that the observed anti-diabetic properties of these plants are mediated, at least partially, through regulating GLUT4 translocation [30].

Glycogen phosphorylase inhibition has been used as one method for treating type 2 diabetes [11,12]. Results of the current study did not show any significant inhibition of glycogen phosphorylase, but extracts of these plants may act on one of other enzymatic reactions that are involved in carbohydrate metabolism and improved glucose homeostasis.

All aqueous extracts showed significantly high toxicity on Brine shrimp Lethality Test, while *Foeniculum vulgare* showed moderate toxicity. Ethanolic extract of *Nigella sativa* showed no toxicity while all other ethanolic extracts exhibited high toxicity. Ethanolic extracts of *Foeniculum vulgare* exhibited the highest toxicity. These statistical consideration are based on the published work by Bussmann and coworkers. They stated that LC_50_ values below 249 μg/ml are considered as highly toxic, 250–499 μg/ml as median toxicity and 500–1000 μg/ml as light toxicity. Values above 1000 μg/ml are regarded as non-toxic [31]. These results could be very useful as preliminary data in the search for new antitumor compounds from the Sudanese market flora. All results for antioxidant activities, glycogen phosphorylase inhibition and cytotoxicity are shown in Table 2.

**Table 2:** Antioxidant activity, enzymatic inhibition and cytotoxicity of selected Sudanese medicinal plants

**Plant**	**Extract**	**DPPH radical scavenging assay %**	**Iron chelating assay %**	**Inhibition % of glycogen phosphorylase (5mg/ml)**	**Brine shrimp lethality (LC **_**50**_**)**
*Acacia Senegal*	Ethanolic	NOT ACTIVE	NOT ACTIVE	0	83.8716
	Aqueous	NOT ACTIVE	NOT ACTIVE	0	17.9948
*Ambrosia maritima*	Ethanolic	60.8 ± 0.04	NOT ACTIVE	2.2	39.7866
	Aqueous	21.2 ± 0.02	NOT ACTIVE	0	10.6353
*Ammi visnaga*	Ethanolic	52.4 ± 0.03	NOT ACTIVE	0	8.1217
	Aqueous	52.4 ± 0.03	2.5 ± 0.03	0	32.6273
*Foeniculum vulgare*	Ethanolic	60.7 ± 0.06	3.6 ± 0.05	0	0.012
	Aqueous	69.4 ± 0.003	NOT ACTIVE	0	893.97
*Nigella sativa*	Ethanolic	47 ± 0.02	6.3 ± 0.02	0	11684.6
	Aqueous	19.3 ± 0.01	43.5 ± 0.04	0	122.268
*Sesamum indicum*	Ethanolic	NOT ACTIVE	NOT ACTIVE	8.2	61.85
	Aqueous	40.3 ± 0.01	23.2 ± 0.02	0	1.7

## Conclusions

In conclusion these results revealed the significant antioxidant activity of the investigated plants extracts and may explain their role in altering the oxidative stress and management of diabetes mellitus. Furthermore the high toxicity of many extracts tested in this study suggests their antitumor potential and provides an avenue to explore the bioactive components of plant extracts. Studies should be directed towards drug industry by identification of single chemical compounds, and dosage use has to be monitored.

### Recommendations

1. In an effort to expand treatment options, there is a need to continue exploring the relationship between free radicals, diabetes, and its complications, and to elucidate the mechanisms by which increased oxidative stress accelerates the development of diabetic complications.

2. Further research into the pathophysiology of oxidative stress and the role of antioxidant therapy will lead to appropriately-designed clinical trials in which the promise of antioxidant therapy will be realized. In addition, further investigations for isolation and identification of active compounds are highly recommended.

3. Extraction processes and usage doses should be monitored. Necessary precautions should be taken while supplementing these extracts in order to avoid other complications of toxicity.

## Competing interest

We declare that we have no competing interests, financially or otherwise.

## Authors’ contributions

YH participated in the study design and coordination, carried out the toxicity assay, drafted the manuscript and rewrote the final one. MA participated in the design of the study, toxicity assay and helped to draft the manuscript. HA conceived of the study, and participated in its design and coordination. AK participated in the enzymatic inhibition study and antioxidant activity. HK supervised part of the study and reviewed the manuscript. All authors read and approved the final manuscript.

## Pre-publication history

The pre-publication history for this paper can be accessed here:

http://www.biomedcentral.com/1472-6882/14/149/prepub
